# Healthy Eating in the Spanish University Community: A Case Study

**DOI:** 10.3390/nu15092053

**Published:** 2023-04-24

**Authors:** Ángeles Arjona Garrido, Montserrat Monserrat Hernández, Juan Carlos Checa Olmos

**Affiliations:** Laboratory of Social and Cultural Anthropology, University of Almeria, 04120 Almeria, Spain; mmh548@ual.es (M.M.H.); jcheca@ual.es (J.C.C.O.)

**Keywords:** food, eating behaviour, Mediterranean diet, University of Almeria

## Abstract

The Mediterranean Diet (MedD), which UNESCO recognizes as an Intangible Cultural Heritage, constitutes a healthy eating pattern that helps prevent illness. The aim of this work is to know how well the university community of Almeria (Spain) adheres to MedD as a healthy lifestyle standard. For this purpose, the authors administered a survey to students, teachers, and administrative and service personnel at the University of Almeria. The sample for the survey comprised 610 people. Of whom, 64.7% were women; 23% were Teaching, and Research Staff (PDI); 17.3% were Administration and Services Staff (PAS); and 59.7% were students. The average age was 32 years. Results show an average level of MedD adherence overall in the university community, although 40.9% have a low adherence level. The most representative MedD adherent can be profiled as a young Spanish female, who values sustainability, reads the labels of the products she consumes, exercises regularly, cooks healthy food, and recycles waste. We suggest [to the University authorities] to advertise the benefits of the Mediterranean Diet among the university community and offer menus based on the MedD in the university canteen.

## 1. Introduction

Eating behavior is influenced by numerous factors that do not always correspond to healthy patterns. Moreover, nutritional value does not tend to be the main motivation for choosing a food product. Cultural, psychological, economic, climatic, social, and geographic factors, among others, mold the dietary profile [1,2,3,4]. A healthy diet protects against nutritional deficiencies and non-communicable diseases: diabetes, heart disease, stroke, and cancer [1], but it requires particular nutritional practices. The Food and Agriculture Organization of the United Nations (FAO) and the World Health Organization (WHO) established dietary standards based on three general assumptions: caloric intake must be balanced with caloric expenditure. Keep salt consumption below 5 g per day (equivalent to less than 2 g of sodium per day). In addition, keep fat consumption to less than 30% of daily caloric intake. Unsaturated fats are preferable to saturated fats. Saturated fats should be <10% of total Kcal and trans fats < 1%. To these, we will add multiple dietary guidelines specific to the trends and habits of the locality (The Spanish Food Safety Agency (AESAN) [5] is the government body in charge of maintaining Spaniards’ food safety) and period of the year.

Widely recommended as healthy, the Mediterranean Diet (MedD) is a culturally transmitted eating pattern consisting of variables such as climate, attitudes, and consumption of certain products [5,6,7]. The Mediterranean Diet was recognized by UNESCO as Intangible Cultural Heritage and a healthy eating pattern by public policy and health professionals [8,9,10]. The MedD emphasizes the consumption of plant-based foods such as vegetables, fruits, whole grains, nuts, and pulses (legumes). The diet also includes protein from small amounts of fish, poultry, and lean cuts of beef. Olive oil is the primary source of fat. In terms of cooking methods, MedD recommends stewing, grilling, or consuming raw vegetables and fruits in salads. Processed sauces, sugar, and salt should be avoided or limited [11]. 

Abundant scientific evidence supports the health benefits of this diet. International research shows that it improves life expectancy and protects against diseases such as Alzheimer’s, cardiorespiratory diseases, cancer, and more [12,13,14,15,16,17,18]. However, studies examining global trends in adherence to MedD show that European countries in the Mediterranean region have experienced a significant decline in adherence over the past 70 years, which has been attributed to the increased consumption of popular (junk) food from Northern Europe and the United States. For instance, Greece, which was at the top of the MedD adherence ranking in the 1960s, has dropped to the tenth position in recent years, while Spain has dropped to the eighteenth position. In contrast, countries such as Denmark have shown improvement, moving from the 40th to the 32nd position, and Morocco has risen to the second position in recent years. North America, on the other hand, has the lowest adherence to the Mediterranean Diet worldwide [19]. This trend is especially pronounced among young people [20]. 

New data show a worldwide rise in unhealthy eating habits [1,2]. The Spanish National Institute of Statistics [15], for example, found that in 2022 young Spaniards between the ages of 15 and 17 consumed a diet high in fast food that requires little preparation, such as sandwiches, pizzas, pasta, hamburgers, meats, etc. Research shows an increase in poor nutrition among young people in recent years. However, during the COVID-19 pandemic, data on the eating habits of young people are unclear. While some studies show healthier habits than previous years [21], others show the opposite [22]. International research on this topic has also reported mixed results [23,24]. This variability in results may be due to several factors, including the phase of the pandemic during which the measurements were taken, the geographical area of residence, and other sociodemographic factors. 

These habits translate into an unhealthy age group of overweight (249,500) and obese (40,600) young people, which represent 16% and 2.6%, respectively, of the 1,530,209 people in the 15–17 age group in Spain [25].

The new life cycle that starts with tertiary education means, for many, leaving the family home, moving to a new city, time pressure, food vendors that offer unhealthy menus, and economic factors that can influence the choice of unhealthy food [26,27], even when the individual internalized healthy eating habits in childhood. The goal of this work is to know the adherence of the university community of Almeria (Spain) to the Mediterranean Diet. According to our hypothesis, sociodemographic factors and consuming habits explain why a high percentage of the university community does not adhere to the Mediterranean diet, particularly students living alone.

## 2. Materials and Methods

Research for this article was carried out at the University of Almeria (UAL) during the academic year 2021/2022 (September–November). Our selection of the UAL community was based on two factors. (1) The community was already interested in the topic. To promote healthy eating habits on campus, the university’s Vice-Rectorate for Sports, Sustainability, and a Healthy University funded the diagnostic survey, “Nutritional Habits, Health, and Environmental Engagement in the University Community”, that we designed, conducted, and analyzed for this paper. (2) This university community belongs to the province of Almeria, where abundant, high-quality food choices are widely available. Often called the orchard of Europe, Almeria is the largest fruit and vegetable producer in Europe, and it provides fresh seafood along 217 kilometers of coastline. Therefore, healthy foods are plenty and at hand in the vicinity of the university campus. 

### 2.1. Participants

The scope of the study covers a university community of 15,268 people. We assumed an error ± 3.9, resulting in 610 completed surveys. Of this sample, 64.7% were women, 34.1% were men, and 1.3% preferred not to say. Further, 23% were Teaching and Research Staff (PDI), and 17.3% were Administration and Services Staff (PAS) (These professionals work in different services: Rectorate, Vice Rectorates, Faculties or Schools, libraries, etc. In addition, they have different professional scales. We grouped them under the heading Management and Administration Services) and 59.7% were students. The average age was 32 years. In total, 92.5% are Spaniards (see Table 1). 

### 2.2. Instrument

The questionnaire, entitled “Nutritional Habits, Health and Environmental Engagement in the University Community”, had four sections: respondents’ informed consent, consumption behavior, food and sustainability knowledge, and lifestyle and sociodemographic data. 

To ascertain the level of adherence to the MedD, we used the Mediterranean Diet Score 2 Tool (T-MDS) [11]. The T-MDS index is calculated by assigning a value of 0 or 1 to nine components of daily intake. A value of 1 is assigned if the respondents’ daily consumption of protective MedD foods is equal to or greater than the average intake of these foods and 0 if consumption is lower. The score evaluates the daily intake of nine components:

- The ratio of monounsaturated fatty acids to saturated fatty acids (MUFA/SFA according to the Spanish Food Composition Database (BEDCA) [28].
- High consumption of vegetables, legumes, fruit and nuts, cereals, and fish.
- Moderate consumption of alcohol, milk, and dairy products.
- Low consumption of meat and derivatives.

To obtain more detailed information on respondents’ eating habits, we also questioned them about the types of foods they consumed in four intake categories: dairy products (skimmed, semi, or whole milk), cereals (whole or white), fish (white or blue), and meat (red or white). 

Following the methods outlined by Giménez [29], we calculated our respondents’ average intake of each dietary element. For each of the components, individuals receive a positive point if their intake is higher than the sample’s average for components understood as “protective” (vegetables, legumes, fruits, nuts, cereals, and fish) and zero if their intake is lower than the average for “non-protective” components (meat and dairy). For the ratio of monounsaturated fats to saturated fats, we scored 1 for above the median and 0 otherwise. 

We assessed the frequency of consumption for each specific type of food or beverage using a table with single-response boxes, coded in appropriate amounts (grams and/or servings), and with eight possible answers (from 0 to 7 days a week). For weekly reference foods and for daily reference foods such as water, dairy, cereals, vegetables, and fruits, we provided 4 possible answers (from 0 to more than 3 servings/day) [30] (To standardize the servings, participants were shown a fact sheet based on the recommended energy and nutrient intake in the European Union, specifying the amount that corresponded to each serving and for each food (for example, 1 serving of white meat = 150 gr; or 1 serving of olive oil = 1 tablespoon) [31]). The reference period about which respondents were questioned was within the last month. 

In general, the sum of points from all components ranges from 0 to 9, where “0” means no adherence to the MedD and “9” means the maximum level of adherence to the MedD. We rated 7–9 points as “good” adherence [17,29,30]. 

### 2.3. Procedure

**Figure 1:**
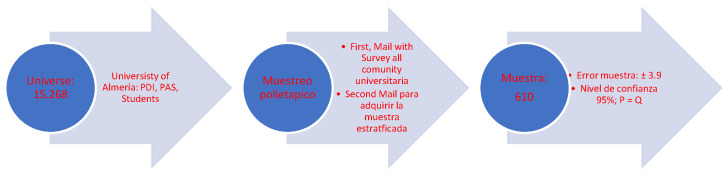
Survey Datasheet. Source: Author’s elaboration.

We obtained our sample in stages. First, we sent an email to the entire university community. Recipients who decided to participate in the study could access the survey through a link attached to the email. Second, we sent another email asking participants to complete a stratified sample according to their role in UAL (Students, Administration and Services Staff, Teaching and Research Staff) [see Figure 1]. 

For data analysis, we used the statistical package SPSS 25.0.0 from IBM Statistics. For the linear regression, we had SPSS convert ordinal and nominal variables into dummy variables, choosing the Faculty of Education Sciences as the SPSS “reference category” for the “study and work center” variable because it had the largest number of UAL students. 

## 3. Results and Discussion

In general, 78.2% of those surveyed considered their lifestyle to be healthy or very healthy, 21.3% unhealthy, and 0.6% not at all healthy. Those who believed that their lifestyle was healthy related it mostly to healthy eating (“eating healthy”, “a varied diet”, “low consumption of fats and sugars”, “following the Mediterranean Diet”) (Spontaneous answers to an open-ended question), along with physical exercise and adequate rest. A total of 71.8% of the university community indicated that they always or almost always maintained a healthy diet, 21.9% sometimes, and 6.2% almost never or never. 

However, in line with the aims of this study, we investigated actual food intake to check the correspondence between respondents’ perception of their healthy eating habits and the parameters for healthy eating as defined by the Mediterranean Diet. Data in Table 2 show that respondents met the recommendations for eating vegetables but were deficient in critical areas: the recommended consumption of cereals, nuts, and seeds, low intake of water and fruit, and excessive consumption of processed foods, salt, and sweets, counter to the recommendations of the AESAN [5]. This could be due to choosing a convenient substitute between meals or snacks. We see a similar outcome in other studies of Spaniards’ eating habits [32,33]. 

When we used these data to test the MedD index, we verified that (1) no one had no adherence at all (value 0) nor total adherence (value 9) (see Table 3); (2) we found the highest percentage (25.1%) for value 1 on the scale. In other words, 1 out of every 4 people in the Almeria University community eats almost no MedD-recommended foods.

**Figure 2:**
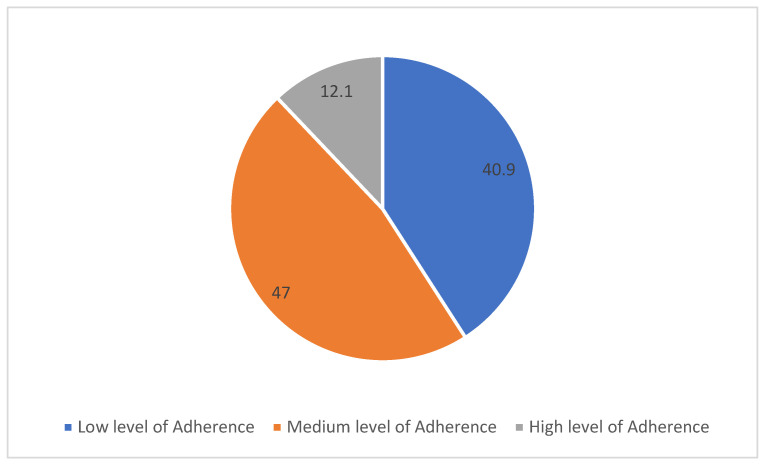
Adherence to the Mediterranean Diet (%). Source: Authors’ elaboration.

More specifically, after grouping the results into three summary blocks, we found that 40.9% had low adherence, 47% had intermediate adherence, and 12.1% had high adherence (see Figure 2).

Collating the level of MedD adherence with respondents’ sociodemographic characteristics, we found that women had a higher adherence rate, 17%, compared to 7.1% for men. Spaniards had higher adherence rates than non-Spaniards, more than half of whom scored at a low level of adherence (see Table 4). 

Taking age into account, we found that no age group showed a high level of MedD adherence. Among people between 41 and 45 years of age, 22.6% was the highest value. The 56 to 60 age group had the lowest, only 5.1%. Therefore, our data mostly showed low adherence, especially among those over 60 years of age and those between 18 and 20 years of age, with medium adherence primarily among those between 51 and 60 years of age (see Table 4).

By role at the university, UAL students showed the lowest adherence to the Mediterranean diet: 42.3% scored low, compared to 27.9% of teaching and research staff and 32.4% of administrative and service staff. 

We found similar results in other studies in Spain [34,35,36,37,38,39,40] and internationally [41,42], which confirm that students consume more fats, proteins, sweets, and snacks than the MedD recommends, and that, as we also observed, they consume lesser quantities of legumes, nuts, fruits, vegetables, and olive oil. This may be due to low economic capital [30], little disposable time for cooking [37], or the novelty of living more independently from their families [35].

Moreover, the lack of MedD adherence among students is not exclusive to Almeria University. We see it in other Spanish universities. At the University of Alicante, for example, no group of students presented a satisfactory intake of the fundamental MedD foods [34]. At the Miguel Hernández University of Elche (Alicante), one in four students had low or very low adherence to MedD [43]. At the University of Granada [27], more than 77% of respondents needed to improve their diets, and only 22.8% had adequate eating habits.

In our opinion, these results are primarily because of the “student lifestyle” and activity-filled university life outweigh education and information about health issues [44]. Second, education alone is not enough without a capacity for analysis and critical thinking. In this regard, the pilot study of Sánchez et al. [38] showed that providing specific information exclusive to MedD led to higher adherence scores. 

However, a few studies present more encouraging data on student adherence. A survey at the Soria campus of the University of Valladolid showed that 11.7% of students had low adherence to MedD (compared to our 42.3%); 46.8%, average; and 41.1%, optimal [43]. In addition, student adherence was generally higher at the University of Extremadura, with an average MedD score of 5.72 [45], compared to 3.9 at the University of Almeria.

**Figure 3:**
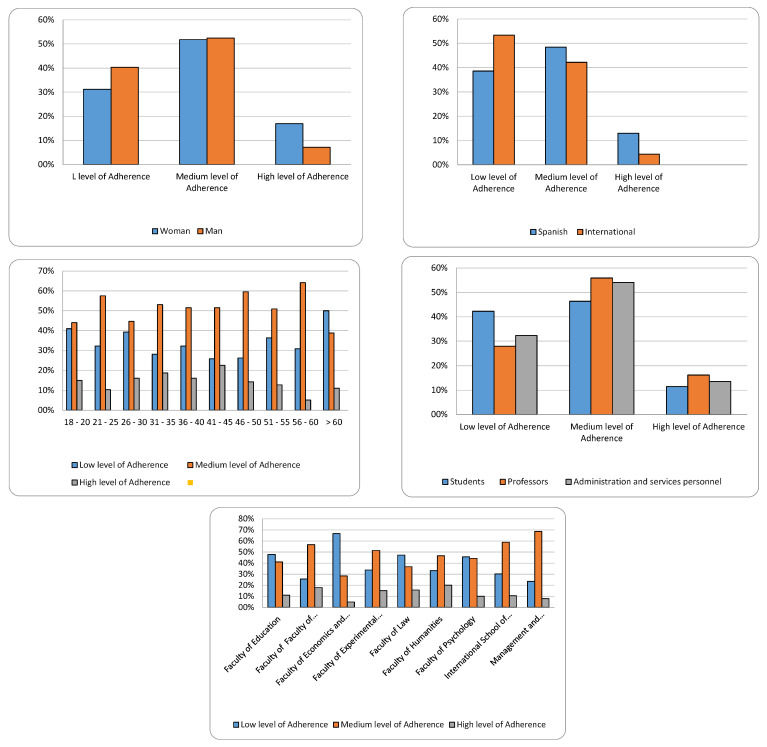
Level of adherence by origin and individual variables (%). Source: Author’s elaboration.

On the other hand, teaching, research, and administrative staff have higher rates of adherence to the MedD than students because of their higher incomes and more structured schedules, which allow them to buy fresh foods in greater variety and schedule time for cooking. By center (studies and work), the Faculty of Humanities has the largest percentage of high MedD adherence (20%), followed by the Faculty of Health Sciences (17.8%). On the low end, the Faculty of Economics and Business Sciences scored only 5% for high-level adherence. Overall, 66.7% showed low adherence to the Mediterranean Diet (see Table 4 and Figure 3). 

To identify possible predictors of MedD variability, we performed four linear regression models (see Table 5):

In Model 1, we introduced individual and demographic variables. Clearly, the most predictive variable is sex (female), followed by Spanish citizenship and, on the negative side, living alone. In other words, Spanish women who live with family members have greater MedD adherence. The variables of student status and young age are negative predictors of MedD adherence. Therefore, the diet of professionals (teaching and research staff, and administrative and service staff) shows the most MedD eating. Youth and being a student correspond to less MedD eating. In short, the combination of sociodemographic variables that predict high scores on MedD adherence suggests the profile of a woman of Spanish nationality, living with her family, not a student, and not young. 

In Model 2, we introduced consumption-habit variables. The first thing we note is that only the personal variable of being a woman has more explanatory weight than consumption habits. Secondly, reading labels and regular exercise are positively related to MedD. Therefore, informing oneself about the products one consumes, avoiding snacks between meals, and regular exercise are the consumption habits that define MedD in our sample.

In Model 3, we added variables of knowledge and information about healthy eating. This allowed us to observe a significant positive relationship between high MedD values and participants’ knowledge of what constitutes a Healthy Dish. Other critical information, however, was not significant in predicting MedD variability, namely, awareness of which foods contain monounsaturated and polyunsaturated fatty acids, knowledge of how to read food labels and knowledge of the glycaemic index and what it tells us about foods.

In this model, personal variables and consumption habits have greater explanatory power for MedD adherence than information about food. 

Finally, in Model 4, we introduced variables of sustainability. We see that throwing food away is a negative predictor of MedD adherence, and recycling waste is a positive predictor. Specifically, people who do not throw food away or recycle waste follow MedD guidelines more closely. 

In summary, Model 4, which encompasses all the variables (individual, consumption habits, Knowledge/awareness, and Sustainability), shows that being a woman and exercising regularly implies greater adherence to MedD, as the international research shows [46,47,48].

In all societies, women are mainly responsible for shopping and food preparation. Women are taught culinary skills and abilities as part of their socialization more often than men, and they are responsible for the culture of beauty [49,50]. As our data confirm, women are more careful with their diet than men. In addition, Spanish women and those living with Spanish relatives—compared to foreign students living away from their family homes—show a higher MedD adherence because of their culinary socialization. The manner of eating with family who shares the same food standards, and the practices of sitting around a table together and carrying on a conversation without digital devices, help to maintain healthy eating patterns [51,52].

Consumption and recreation habits also factor into the MedD score. Consumers who read labels to find out about the characteristics of what they eat show more interest in healthy food practices, such as MedD. 

The relationship between regular physical activity and adherence to MedD is consistent with the other relationships we have pointed to as indicators of healthy living. Alone, exercise does not necessarily predict healthy eating. Physical activity is the greatest benefit to a healthy life when the motivation to exercise is intrinsic rather than extrinsic [53], that is, exercising for personal enjoyment and satisfaction, as opposed to seeking an external benefit or reward [54]. Survey data from Padial, Viciana, and Palomares [27] suggest a case of extrinsically motivated exercise, when 77% of their sample reported nutritional deficiencies, although 86.6% exercised regularly. 

## 4. Conclusions

The primary conclusion we derive from our results is that a significant number of people at the University of Almeria do not comply with the recommendations for healthy eating issued by competent authorities. 

The second conclusion concerns student status at UAL. Of all the participant roles we tested, students are the least adherent to MedD. In our case study, academic education had little relevance to the level of MedD adherence. Even students of the Faculty of Health Sciences and the Faculty of Education, which offers degrees in Physical Education and Sports, did not show high adherence to MedD compared to other faculties. 

As a third conclusion, the linear regression shows two personal variables—(1) Spanish women and (2) living with families and children—to have the greatest effect on the variability of MedD adherence. These two variables consistently predict high values for three critical indicators: healthy consumption habits, regular exercise, and a sustainable lifestyle (not wasting food and recycling). 

### Intervention Plan

Despite the detailed data on the Almeria University community’s average level of MedD adherence, and due to the benefits that high MedD levels generate, we believe that intervention in food consumption is required in order to improve the health standards of the university community. We propose two major lines of work: 

First, we must disseminate knowledge on healthy habits beyond the instruction currently received by students of Health Sciences and Physical Activity and Sports. Regardless of the role a person performs on the university campus, adequate knowledge of healthy eating and the implications of that knowledge for everyday life will facilitate an increase in MedD adherents and the transmission of adherence through their social relationships, both university, and family. 

Second, we must intervene in the offerings of the many catering services on campus to encourage MedD-recommended food choices that utilize local products. As noted above, Almeria supplies the whole of Europe with fruit and vegetable products and has many fishing ports. Thus, instead of advertising unhealthy, ultra-processed foods, the university should publicize the benefits of MedD more widely.

This intervention began in an incipient way during the academic year 2022/2023 with the creation of a registered action protocol [55]. Its effects will be assessed in future studies. 

## Figures and Tables

**Table 1:** Sample characteristics.

		%
Sex	Women	64.7
	Men	34.1
Origin	Spanish	92.5
	International	7.5
Role or profession	Students	59.7
	Professors	23
	Administration and services personnel	17.3
Work or study center	School of Engineering	6.4
	Faculty of Education	14.8
	Faculty of Health Sciences	14.8
	Faculty of Economics and Business Sciences	9.8
	Faculty of Experimental Sciences	14.1
	Faculty of Law	6.2
	Faculty of Humanities	4.9
	Faculty of Psychology	11.5
	International School of Doctoral Programmes	9.2
	Management and Administration Services	8.4

Source: Authors’ elaboration.

**Table 2:** Relationship between MedD recommendations and survey results.

Food Group	Servings Recommended (BEDCA *)	% Surveyed Who Follow Recommendations (Total)
Water	1.5 to 2 L of water per day.	8.8%
Vegetables	Minimum 2 servings per day.	90.7%
Cereals	1–2 servings per day, preferably the whole-grain variety.	42.2%
Olive oil	2–3 servings per day.	56.4%
Fruit	2–3 servings per day.	24.7%
Dairy products	2–3 servings per day.	63.8%
Nuts and seeds	1–2 servings per day.	67.6%
Fish	2–3 servings per week. **	64.5% oily fish; 83.4% white fish
White meat	2 servings per week.	58.2%
Red meat	No more than 2 servings per week.	93.55%
Salt	To reduce consumption or avoid it by substituting it with spices.	46%
Processed food	Less than once per week.	40.2%
Sweets	Less than 2 servings per week.	23.9%
Wine	With moderation and respecting customs.	46%

* BEDCA (Base Española de Datos y Composición de Alimentos). ** The recommended guidelines do not distinguish between blue or white fish (this study separated them due to curiosity about the consumption of polyunsaturated fatty acids in the population surveyed). Source: Authors’ elaboration.

**Table 3:** Adherence to the Mediterranean Diet.

Score	*N*	%
1.00	153	25.1
2.00	34	5.6
3.00	62	10.2
4.00	92	15.1
5.00	105	17.2
6.00	90	14.8
7.00	48	7.9
8.00	26	4.3
Total	610	100.0

Source: Authors’ elaboration.

**Table 4:** Level of adherence by origin and individual variables (%).

		Low Level of Adherence	Medium Level of Adherence	High Level of Adherence
Sex	Woman	31.2%	51.8%	17.0%
	Man	40.4%	52.5%	7.1%
Origin	Spanish	38.6%	48.4%	13.0%
	International	53.3%	42.2%	4.4%
Age	18–20	41.0%	44.0%	15.0%
	21–25	32.2%	57.5%	10.3%
	26–30	39.3%	44.6%	16.1%
	31–35	28.1%	53.1%	18.8%
	36–40	32.3%	51.6%	16.1%
	41–45	25.8%	51.6%	22.6%
	46–50	26.2%	59.5%	14.3%
	51–55	36.4%	50.9%	12.7%
	56–60	30.8%	64.1%	5.1%
	>60	50.0%	38.9%	11.1%
Role or activity	Students	42.3%	46.3%	11.5%
	Professors	27.9%	55.9%	16.2%
	Administration and services personnel	32.4%	54.1%	13.5%
Centre of study or work	School of Engineering	64.1%	28.2%	7.7%
	Faculty of Education	47.8%	41.1%	11.1%
	Faculty of Health Sciences	25.6%	56.7%	17.8%
	Faculty of Economics and Business Sciences	66.7%	28.3%	5.0%
	Faculty of Experimental Sciences	33.7%	51.2%	15.1%
	Faculty of Law	47.4%	36.8%	15.8%
	Faculty of Humanities	33.3%	46.7%	20.0%
	Faculty of Psychology	45.7%	44.3%	10.0%
	International School of Doctoral Programmes	30.4%	58.9%	10.7%
	Management and Administration Services	23.5%	68.6%	7.8%

Source: Authors’ elaboration.

**Table 5:** Models explaining the degree of MedD.

Personal Variables		Model 1	Model 2	Model 3	Model 4
	Sex (female)	0.295 *	0.257 *	0.254 *	0.264
	Household type (alone)	−0.144 **	−0.123 **	−0.096 **	−0.103
	Origin (Spanish)	0.179 *	0.148 *	0.154 *	0.141
	Role (student)	−0.097 **	−0.083 **	0.044 ***	0.038
	Age	−0.054 **	−0.050 **	−0.155 **	−0.141 **
	School of Engineering	0.065 ***	0.065 ***	0.064 ***	0.067
	Faculty of Health Sciences	0.011 ***	0.039 ***	0.030 ***	0.064 ***
	Faculty of Economics and Business Sciences	0.106 ***	0.131 ***	0.135 ***	0.162 ***
	Faculty of Experimental Sciences	−0.004 ***	−0.011 ***	0.003 ***	0.27 ***
	Faculty of Law	−0.069 ***	−0.037 ***	−0.044 ***	−0.029 ***
	Faculty of Humanities	0.019 ***	0.040 ***	0.035 ***	0.054 ***
	Faculty of Psychology	−0.090 ***	−0.042 ***	−0.036 ***	−0.035 ***
	International School of Doctoral Programmes	−0.006 ***	−0.044 ***	−0.056 ***	−0.045 ***
	Management and Administration Services	−0.058 ***	−0.043 ***	−0.040 ***	−0.034 ***
Consumption habits	Read the labels		0.159 *	0.155 *	0.129 *
	I like cooking		0.097 **	0.089 **	0.095 **
	Snacking between meals		−0.123 **	−0.128 **	−0.122 **
	Plan a meal		0.030 ***	0.026 ***	0.016 ***
	Regular exercise		0.149 *	0.140 *	0.132 *
Knowledge/ awareness	Healthy Dish			0.123 **	0.015 **
	Omega-rich salmon			0.084 ***	0.080 ***
	High GI egg			0.019 ***	0.015 ***
	Healthy Labelling			0.041 ***	0.024 ***
Sustainability	Throwing food away				−0.108 **
	Interest in learning about the environment				0.007 ***
	Recycle waste				0.121 **
	Type of bags I use				−0.92 ***
Coefficient/ratio R^2^		0.241	0.309	0.318	0.344

Source: own elaboration. * *p* < 0.01; ** *p* < 0.05; *** *p*> 0.05.

## Data Availability

Not applicable.
